# A biodynamic model predicting copper and cadmium bioaccumulation in caddisflies: Linkages between field studies and laboratory exposures

**DOI:** 10.1371/journal.pone.0297801

**Published:** 2024-02-22

**Authors:** Michelle I. Hornberger

**Affiliations:** U.S. Geological Survey, Menlo Park, CA, United States of America; Universidade Regional Integrada do Alto Uruguai e das Missoes, BRAZIL

## Abstract

*Hydropsyche* and *Arctopsyche* are filter-feeding caddisflies (Order: Trichoptera; Family: Hydropsychidae) that are commonly used to monitor metal exposures in rivers. While tissue residue concentrations provide important bioaccumulation data regarding metal bioavailability, they do not provide information regarding the mechanisms of uptake and loss, or exposure history. This study examined the physiological processes that control Cu and Cd uptake and loss using a biokinetic bioaccumulation model. Larvae of each taxon were experimentally exposed to either water or food enriched with stable isotopes (^65^Cu and ^106^Cd). Dissolved Cu uptake (k_u_) was similar between species (2.6–3.4 L^-1^g ^1^d^-1^), but Cd uptake was 3-fold higher in *Hydropsyche* than *Arctopsyche* (1.85 L^-1^g ^1^d^-1^ and 0.60 L^-1^g ^1^d^-1^, respectively). Cu and Cd efflux rates (k_e_) were relatively fast (0.14 d^-1^–0.24 d^-1^) in both species, and may explain, in part, their metal tolerance to mine-impacted rivers. Food ingestion rates (IR), assimilation efficiency (AE) of ^65^Cu and ^106^Cd from laboratory diets were also derived and used in a biodynamic model to quantify the relative contribution of dissolved and dietary exposure routes. Results from the biodynamic model were compared to tissue concentrations observed in a long-term field study and indicated that because dissolved Cu and Cd exposures accounted for less than 20% of body concentrations of either taxon, dietary exposure was the predominant metal pathway. An estimation of exposure history was determined using the model to predict steady state concentrations. Under constant exposure conditions (dissolved plus diet), steady state concentrations were reached in less than 30 days, an outcome largely influenced by rapid efflux (k_e_).

## 1. Introduction

Metal body burdens in aquatic taxa are widely used in place of, or in combination with, environmental measurements to determine metal occurrence [1, 2]. Field studies using freshwater insects as biomonitors have linked biological effects related to metal exposure from both legacy [3–6] and active [7–9] mining activities. Aquatic insects are relatively sessile, geographically widespread and have well characterized life-history patterns that provide ecological context when interpreting bioaccumulation data. And while water quality and bed sediment criteria provide an important baseline to assess stream health, biological effects can only be inferred. Metal accumulated into the tissues of resident aquatic organisms are a direct indicator of bioavailability and environmental condition [1].

Empirical approaches to monitoring metal bioavailability–e.g. measuring body burdens in a widely dispersed taxon–provide biological evidence of metal exposure, these approaches do not address the fundamental processes driving bioaccumulation and predictive capability [10]. For these reasons, experimental studies have been important in parameterizing the physiological kinetics bioaccumulation models that can help explain observational data and simulate metal bioaccumulation under measured site-specific geochemical conditions [11].

The biodynamic model considers basic physiological processes that affect metal bioaccumulation [12, 13]. The model has been applied to address fundamental questions regarding metal bioaccumulation patterns, including the relative contributions of aqueous and dietary metal exposure to body burdens [14–18], metal-specific bioaccumulation [19, 20], species-specific bioaccumulation [21, 22], and site-specific predictions of metal bioaccumulation by endemic and introduced taxa [23, 24]. Studies focused on aquatic insects typically rely on two premises. First, metal uptake from ingested matter is the dominant exposure pathway for primary consumers [22]. This follows from earlier studies that linked tissue metal concentrations to feeding traits [25]. Hence, food selectivity influences species-specific metal body burdens. Second, physiological traits relevant to aqueous metal uptake and efflux appear to be phylogenetically related [26]. Both constants tend to be more similar among closely related taxa (e.g., within families and genera) than to more distantly related taxa. Therefore, species-specific differences may be expected to vary with phylogenetic distance if aqueous uptake and elimination are the dominant drivers of metal bioaccumulation.

This study examines the mechanisms affecting metal bioaccumulation in two filter-feeding caddisflies, *Hydropsyche* and *Arctopsyche*. Both genera are members of the Hydropsychidae [27] feeding largely on seston, although *Arctopsyche* becomes more predaceous in later instars [25]. Both species bioaccumulate metals proportionally to environmental levels of metal contamination [28–31]. This characteristic along with their ubiquitous distribution, sessile habit, and typical one year life span (univoltine) makes them useful metal biomonitors [28]. However, where the taxa coexist, differences in metal body burdens have often–but not always–been observed. Previous studies have reported on dissolved Cd [32], and Cu [33] exposure kinetics for *Hydropsyche*, but comparable data for *Arctopsyche* are not available. This study uses a novel stable isotope tracing technique [34] to derive physiological constants for a biodynamic model which considers both dissolved and dietary exposures. By incorporating laboratory derived physiological parameters of metal uptake and loss, predictions of metal body burden can be determined and used to improve our understanding of field biomonitoring data. The model is used to address three objectives: 1) quantify the relative importance of dissolved and dietary exposure pathways; 2) identify the influence of physiological processes influencing metal bioaccumulation; 3) address the implications of the results to biomonitoring studies.

## 2. Materials and methods

### 2.1. Experimental organisms

Both *Arctopsyche* and *Hydropsyche* belong to the family Hydropsychidae (O: Trichoptera). They are relatively sessile, net-spinning filter feeders and while both are categorized as omnivores, *Arctopsyche* are more predatory than *Hydropsyche* [25, 35]. They often co-occur and both are generally univoltine (although see Smith, [36]). Because of these characteristics, both species are useful biomonitors in metal contaminated rivers. Specific details on field handling and collection are described elsewhere [37, 38]. Briefly, *Hydropsyche* experiments were conducted with *Hydropsyche californica* collected from Stevens Creek, California in 2006 and 2008 (37°18’24.35"N, 122° 4’22.02"W). *Arctopsyche* experiments were conducted with *Arctopsyche grandis* collected from Rock Creek, Montana in 2010 and 2012 (46°41’57.25"N, 113°40’2.34"W). Neither site has a history of metal contamination. Approximately 150 organisms of 4^th^-5^th^ instar were collected for each experiment. Experiments were designed to minimize metal complexation in solution, so natural stream water was progressively replaced with artificial very soft river water (VSW, hardness 10–13 mg CaCO_3_ L^-1^ [39]) over a 3-day acclimation period. Experiments were conducted in a constant temperature room, maintained at 15°C and pH at 6.5–6.7 for all experiments.

### 2.2. Biodynamic model

The rate of change in an organism metal concentration [M] (μg g^-1^, dry wt) can be expressed as the difference between metal entering and leaving the organism per unit time (d) if metal dilution due to animal growth is considered [40, 41]. That is,

$$d{\left[M\right]}_{t}/d_{t}=k_{u}[M_{w}]+(AE\cdot IR\cdot [M_{f}])–(k_{e+g}){\left[M\right]}_{t}$$ (1)

where k_u_, (L g^-1^d^-1^) is the conditional uptake rate constant from solution, [M_w_] (μg L^-1^) is the metal concentration in water, AE is the metal assimilation efficiency, IR (g g^-1^ d^-1^) is the food ingestion rate, [M_f_] (μg g^-1^) is the metal concentration in food and k_e+g_ (d^-1^) are the combined rate constants for metal efflux and body growth dilution. Short term pulse-chase experiments are often used to parameterize these physiological rate constants. Because of the short duration of the experiments (Table 1) compared to the lifespan of the organism, growth can usually be ignored. Thus, the integrated form of Eq 1 becomes:

$${\left[M\right]}_{t}=(k_{u}[M_{w}]+(AE\cdot IR\cdot [M_{f}]))/k_{e}\cdot (1‐e^{‐ket})$$ (2)

At steady state, (i.e., *d*[M]/*d*_t_ = 0), Eq 1 can be simplified to:

$${\left[M\right]}_{ss}=(k_{u}[M_{w}]+AE\cdot IR\cdot [M_{f}])/k_{e}$$ (3)

where [M]_ss_ is the metal concentration a steady-state (μg g^-1^, dry wt).

**Table 1:** Experimental conditions used to determine physiological parameters for caddisflies in the biodynamic model. Dissolved 65Cu and 106Cd represent nominal total dissolved concentrations. All experiments conducted using very soft synthetic river water [39]. Average ± SD listed for dry weights and metal concentrations.

Experiment Model Parameters	Experimental Conditions	*Arctopsyche*	*Hydropsyche*
**Dissolved Uptake (k**_**u**_ **)**	Exposure (hour)	8	24
	^65^Cu, μg L^-1^	Control, 0.5, 1.5, 4.5, 13.5	Control, 1, 10, 100, 300
	^106^Cd, μg L^-1^	Control, 0.1, 0.3, 0.9, 2.7	Control, 0.1, 1.0, 10, 30
	Organism dry wt (mg)	5.1 ± 0.8	6.0 ± 1.2
**Dietary Uptake (AE, IR, [M]**_**f**_ **)**	Exposure (hour)	8	8
	Treatment Food	*Lymnaea stagnalis*	*Lumbriculus variegatus*
	^65^Cu_*treatment*_, μg g^-1^	42.0 ± 4.4	104 ± 3
	^106^Cd_*treatment*_, μg g^-1^	12.3 ± 0.6	21.8 ± 0.4
	Depuration (hour)	24	24
	Organism dry wt (mg)	4.8 ± 0.5	2.1 ± 0.3
**Loss (k**_**e**_ **)**	^65^Cu, μg L^-1^	100	100
	^106^Cd, μg L^-1^	5	30
	Depuration (days)	9	19
	Organism (mg dry wt)	7.3 ± 1	6.1 ± 0.4

### 2.3. Bioaccumulation experiments

Dissolved uptake rate constants (k_u_) were determined from the slope of the linear relationship between metal influx (μg g^-1^ d^-1^) and the measured total dissolved and free-ion metal concentrations (μg L^-1^ d^-1^). Accumulated tracer concentrations in tissues were determined as described by Croteau and Luoma [42]. Briefly, treatments were simultaneously spiked with commercially purchased stable isotope standards (Trace Sciences International) enriched with ^65^Cu (99.4%) and ^106^Cd (96.5%) to produce a range of dissolved and dietborne exposure conditions (Table 1). Signal intensities in the ICPMS calibration standards were used to determine the proportional abundance of each tracer *p*^*i*^ (^106^Cd = 0.011, ± 0.001; ^65^Cu = 0.359 ± 0.03, n = 4). Concentrations (μg g^-1^) of ^106^Cd and ^65^Cu in the experimental organisms ([^*i*^E]_ê_) were calculated by multiplying *p*^*i*^ by the total metal concentration (μg L^-1^) inferred from the tracer intensity ([T^*i*^E]):

$${{[}^{i}E]}_{ê}=p^{i}\;x\;[T^{i}E]\;x\;Vol÷DW$$ (4)

Where Vol is reconstituted volume (mL) of the digested sample and DW is the dry weight (g) of the organism. The pre-existing concentration of the tracer, [^*i*^E]^0^_ê_ was determined by:

$${[}^{i}E{{]}^{0}}_{ê}=p^{i}x\;[T^{k}E]\;x\;Vol÷DW$$ (5)

where [*T*^*k*^*E*] is concentration of the most abundant isotope. The newly accumulated tracer concentration was calculated by subtracting ([T^*i*^E]–[T^k^E]).

Speciation calculations using WHAM 6.0 [43] indicated that respectively, 90% and 97% of the total added Cu and Cd occurred as free ions, the fraction most bioavailable to aquatic organisms. The experimental chambers were conducted in acid-washed (15% nitric and 5% hydrochloric) high-density polyethylene (HDPE) containers and continually aerated using air pumps and acid-washed tubing [37]. An artificial substrate consisting of one or two 15 cm^2^ pieces of nylon mesh was inserted into each experimental container. To minimize surface adsorption on experimental surfaces, containers and net substrates were soaked and rinsed with the spiked media prior to the exposure. Organisms were exposed to a range of dissolved metal concentrations (Table 1) for 8 (*Arctopsyche*) or 24 hours (*Hydropsyche*) in the absence of food. The exposure range and duration were selected independently for each species to account for potential species-specific sensitivity of metal partitioning in cytosolic proteins [5]. After exposure, 10–12 individuals and three filtered water samples (acidified to 2%, Baker Ultrex II grade HNO_3_) were collected from the control and each treatment. No mortalities were observed during the dissolved exposure period.

Dietary uptake experiments were conducted in acid-washed 5 L aquaria using modified Trent Tubes [44]. A three-day acclimation period was used so that the larvae could adjust to both the media (as described above) and physical setting, e.g., organisms roamed freely inside the screened-off tube and built “retreats” (a shelter anchored to a substrate). A current oriented towards one end of the tube was created using a submersible pump. The caddisflies aligned their retreats with the current to trap food on their silk nets (S1 Fig). After the acclimation period, organisms were fed either tracer-enriched food (treatment, as described below) or non-labeled control food for 8 hours (Table 1). At the end of the feeding period, the caddisflies (n = 12 *Hydropsyche*; n = 14 *Arctopsyche*) were transferred to individual artificial retreats placed in a 38 L, trace metal clean depuration aquaria, filled with 20 L of artificial VSW for 24 hours. After the depuration period, each larva was collected, rinsed with VSW, placed in an acid-washed vial and frozen. Water samples were collected before and after feeding, and after depuration (data reported in Hornberger and Croteau, 2023 [38]).

The food used during the exposure treatments consisted of dried and finely ground laboratory cultured freshwater snails (*Lymnaea stagnalis*) and oligocheates (*Lumbriculus variegatus*), each pre-exposed to dissolved metals. While this food differs from the natural diet of the two caddisflies, snails and worms accumulate metals from the aqueous phase [42, 45], enabling the preparation of food items with internalized labels instead of surface-bound tracers. Two to three milligrams of homogenized tissue mixed in artificial VSW (5–10 ml) were dispersed into the current using a transfer pipette. *Hydropsyche* and *Arctopsyche* were fed at 2-hr increments throughout the 8-hr period, although food continued to circulate through the chamber. After 8 h, each organism was transferred to the 38 L depuration aquarium (as described above) for 24 hours. The residence time of food in the gut of caddisfly larvae can range from 12–72 hours [46–48]. After depuration, animals were removed, and their feces were collected by filtration (1.2 μM membrane filter). Samples were immediately frozen prior to metal analysis. Water samples (n = 3, acidified to 2% with ultrex HNO_3_) were collected before and after exposure, as well as the end of the depuration period. Samples collected after the exposure and depuration periods were filtered with a 0.45 μM Pall^®^ membrane filter.

Assimilation efficiency (AE, unitless) and ingestion rate (IR, g g^-1^ d^-1^) were determined by mass balance calculations, i.e.,

$$AE=M_{r}/(M_{r}+M_{f})$$ (6)

where M_r_ is the amount (ng) of tracer retained in the organism after the 24-hr depuration period, and M_f_ is the amount (ng) of tracer in the feces.

IR (g/g/d) is a function of the ingested tracer (M_r +_ M_f_), the metal concentration in the food [M_f_], the weight of the organism (Wt, mg), and feeding time (T, day):

$$IR=(M_{r}+f)/[M_{f}]•Wt•T$$ (7)

Calculations were performed on each individual caddisfly within a treatment (or control). Ingestion rates were determined for each tracer. The averages of the two IR estimates (±95% CI) are reported here.

Efflux experiments were conducted by exposing organisms to dissolved tracer concentrations for up to 2 days and then transferring them to clean artificial VSW for up to 19 days. Caddisflies were fed daily a mixture of the non-labeled diet during the depuration phase. The *Hydropsyche* efflux experiment for lasted 19 days, with 9 individuals collected on days 0, 1, 2, 4, 6, 9, 12 and 19. Due to increasing mortality, the depuration time for *Arctopsyche* was limited to 9 days, with 9 individuals collected on days 0, 1, 2, 3, 5, 7 and 9. Filtered (0.45 μm) water samples (n = 3) were collected each day and acidified to 2% with Ultrex HNO_3_.

Efflux rate constants (k_e_) were determined from the relationships between the proportional loss of each tracer over time. Loss was assumed to follow a one-compartment model starting day 1 and was calculated by nonlinear regression

$$M_{t}/M_{0}=a•exp^{\left(‐ke•t\right)}$$ (8)

where M_*t*_ is the metal concentration in the larvae at time t, M_0_ is the mean concentration at the beginning of the loss phase (t_0_), *a* is the proportion of accumulated tracer after the exposure phase and *t* is the days of depuration.

### 2.4. Sample preparation and analysis

Metal analyses for all samples followed the protocol outlined by Croteau et al. [34]. Briefly, samples were oven-dried at 50°C for 1–3 days, cooled to room temperature and weighed. Samples were placed in an acid washed Teflon^®^ sealed container. Ultrex HNO_3_ (100 μL per mg dry weight sample) was added to each container and stored at room temperature for 7 days. Hydrogen peroxide (Baker Ultrex II grade, 40 μL per mg dry weight sample) was then added for 24 hrs. Samples were filtered (<0.45 μm) and diluted with double-deionized water (>18 Mohm cm^-1^) to a total volume of 3 mL.

All samples were analyzed using an Elan 6000 Inductively Coupled Plasma-Mass Spectrometer (ICP-MS, single-detector quadrupole by Perkin Elmer). Two replicate exposures consisting of 32 individual measurements for each exposure were analyzed for each tissue and water sample [34]. All samples and standards were spiked with Ge (8 μL per mL of sample) as an internal standard to account for instrument drift. Accumulated ^65^Cu and ^106^Cd tracer concentrations were determined using the method described by Croteau and Luoma [42]. Riverine water reference material (SLRS-4) and calibration standards were analyzed throughout each run [38].

### 2.5. Quality control and statistical analysis

Standard Reference Materials (SRM 2976, mussel tissue; TORT-2, lobster hepatopancreas) and procedural laboratory blanks were digested using the procedures described above. Copper and Cd percent recoveries for mussel tissue SRM 2976 (Cu, 98.8% ± 9.5 SD; Cd, 87.6% ± 12.3 SD) and lobster hepatopancreas TORT-2 (Cu, 89.1% ± 4.9 SD; Cd, 85.7% ± 10.6 SD) were within the 95% confidence interval of the certified values and are reported in Hornberger and Croteau [38]. Because data were not normally distributed, a Mann-Whitney U test was used to test for species differences in ingestion rates and assimilation efficiencies. Analysis of covariance (ANCOVA) was used to test for homogeneity of slopes for uptake and loss rates. Piecewise regression was used to identify the period between uptake and steady state. Statistical analysis was conducted using Statistica v.12 (Statsoft, 2013) and all significance levels were α = 0.05.

## 3. Results

### 3.1. Dissolved and dietary metal uptake

**Fig 1:**
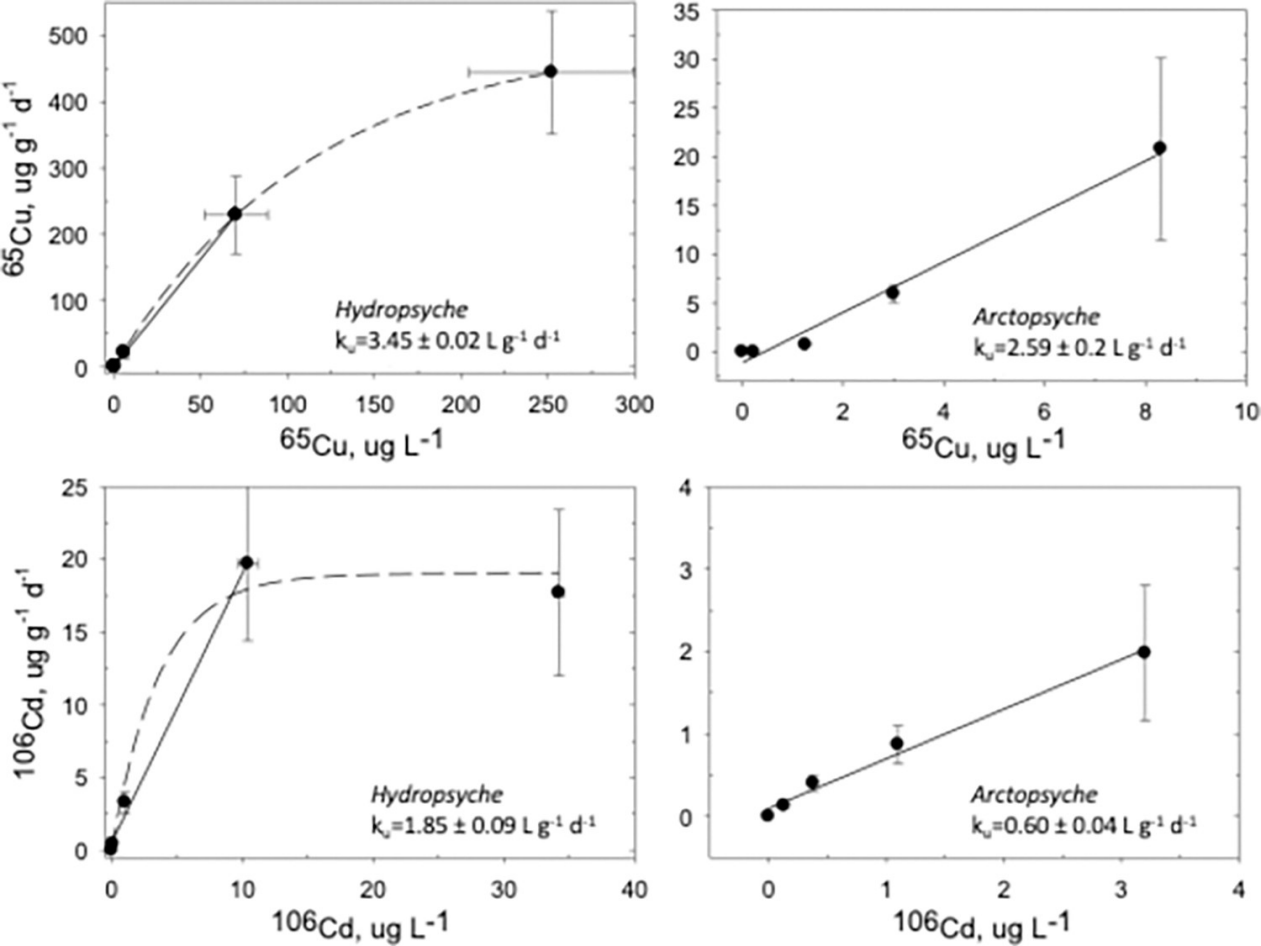
Dissolved ^65^Cu and ^106^Cd uptake rates (k_u_, mean ± 95% CI, L g^-1^d^-1^) for *Hydropsyche* and *Arctopsyche*. Dissolved exposure for the enriched stable isotope tracers ^106^Cd and ^65^Cu (x-axis, μg L^-1^) are plotted against *Hydropsyche* and *Arctopsyche* influx rates (y-axis, μg g^-1^ d^-1^). Uptake rates were determined by linear regression and were based on data within the linear range of influx (see methods). The dashed line is the non-linear regression exponential fit for all treatment concentrations for *Hydropsyche*. Saturation was not observed in *Arctopsyche*.

Copper and Cd dissolved influx rates in *Hydropsyche* and *Arctopsyche* increased with increasing total dissolved metal concentrations (Fig 1). Specifically, Cu uptake rates in *Hydropsyche* increased linearly up to 75 μg L^-1^ and became non-linear at exposure concentrations higher than 75 μg L^-1^ (Fig 1). Similarly, Cd influx rates in *Hydropsyche* appeared linear up to exposure concentrations of approximately 10 μg L^-1^ and remained comparable at higher concentrations (Fig 1). *Arctopsyche* influx rates for both metals were linear across the range of exposure concentrations (although *Arctopsyche* exposure concentrations were lower than experiments conducted with *Hydropsyche*). Despite the differences in exposure concentrations between species, the rate constants for Cu uptake (mean ± 95% CI) were not significantly different (ANCOVA *P* = 0.294) between *Hydropsyche* and *Arctopsyche* (k_u_ = 3.4 ± 0.02 L g^-1^ d^-1^; 2.6 ± 0.20 L g^-1^ d^-1^, respectively). In contrast, the Cd influx rate constant for *Hydropsyche* was significantly higher (ANCOVA, *P* = 0.001) than *Arctopsyche* (k_u_ = 1.8 ± 0.1 L g^-1^ d^-1^; vs. 0.60 ± 0.04 L g^-1^ d^-1^, respectively).

Caddisfly Cu and Cd assimilation efficiency (AE) ranged from 70–96%, with *Hydropsyche* AE higher than *Arctopsyche* (Table 2, *P* = 0.002 and *P* = 0.01, respectively). Ingestion rates (IR) were also higher in *Hydropsyche* (0.07 vs. 0.03 g g^-1^ d^-1^, *P* = 0.005, Table 2). The lower IRs in *Arctopsyche* may be caused by limited food intake of the experimental diet due to their predaceous traits or indicate stress related feeding inhibition.

**Table 2:** Laboratory derived parameters for the biodynamic model (mean values ± 95% CI).

		Copper		Cadmium	
Parameter	Unit	*Hydropsyche*	*Arctopsyche*	*Hydropsyche*	*Arctopsyche*
Dissolved Uptake (k_u_)	L g^-1^ d^-1^	3.45 ± 0.02	2.59 ± 0.2	1.85 ± 0.09	0.60 ± 0.04
Efflux (k_e_)	d^-1^	0.17 ± 0.01	0.18 ± 0.04	0.24 ± 0.01	0.14 ± 0.06
Assimilation Efficiency (AE)	%	93 ± 5	70 ± 15	96 ± 1	86 ± 7
Ingestion Rate (IR)	g g^-1^ d^-1^	0.07 ± 0.02	0.03 ± 0.01	0.07 ± 0.02	0.03 ± 0.01

Dissolved tracer concentrations increased from <0.003 μg L^-1^ up to 6.7 μg L^-1^ (ppb) over the 8-hour feeding period, likely due to the ongoing resuspension of spiked food in the chamber. However, because these concentrations are orders of magnitude lower than dietary concentrations (Table 1, ppm), uptake via dissolved exposure during the dietary exposure experiments was assumed to be negligible.

### 3.2. Efflux kinetics

**Fig 2:**
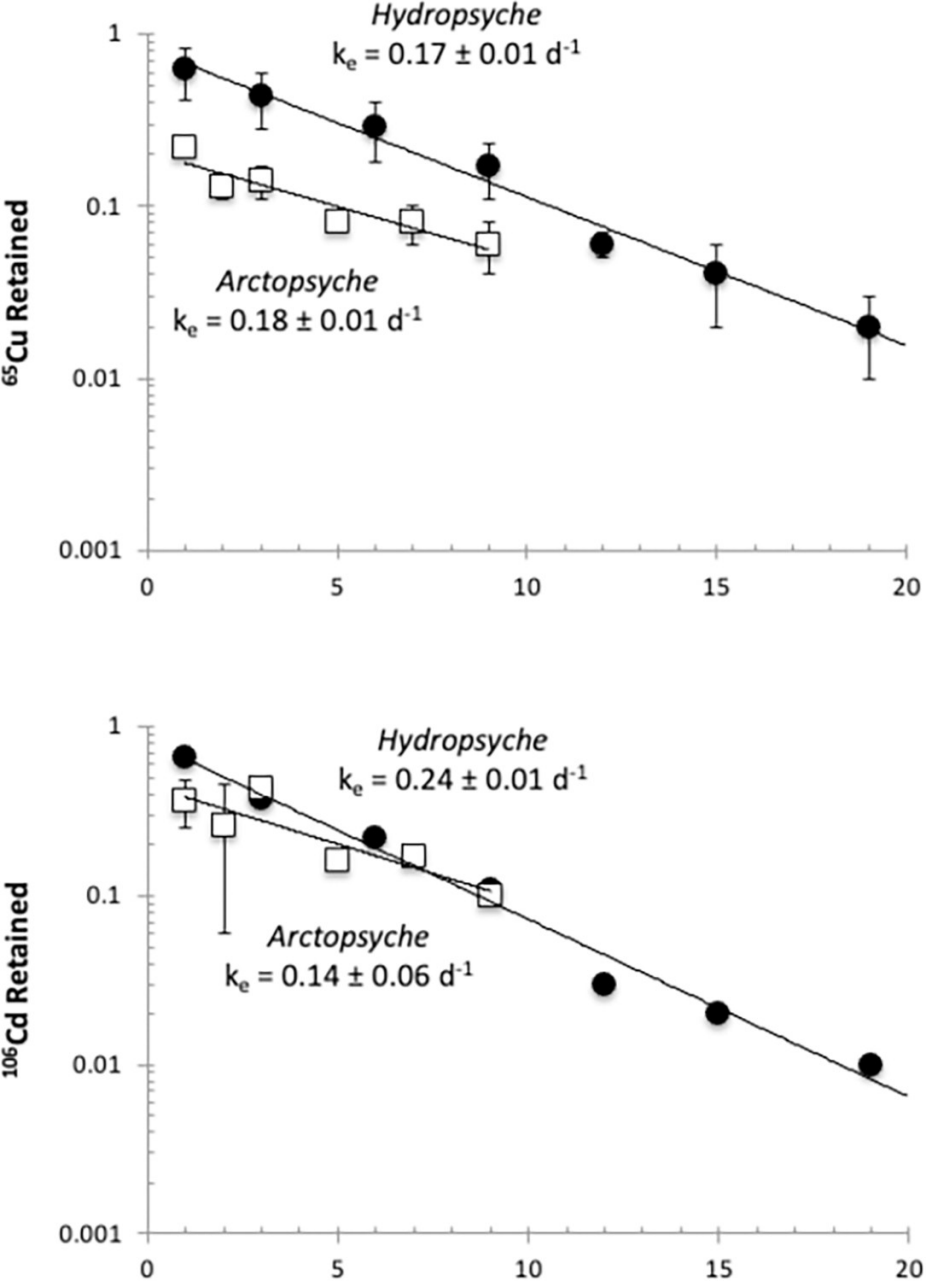
Efflux rates (k_e,_ mean ± 95% CI, d^-1^) of ^65^Cu and ^106^Cd for *Hydropsyche* (black circles) and *Arctopsyche* (gray squares) determined by linear regression (solid line): Proportion of tracer retained (%) plotted against day.

The Cu and Cd efflux rate constants (k_e_) were similar in both species of caddisflies, each losing between 35–70% of the tracers within the first 24 hours (Fig 2). Because this initial loss may be due to excretion and/or desorption of loosely bound metal on the body surface [22], the loss during these first 24 h of depuration was excluded from the k_e_ calculation. Approximately 18% of the remaining Cu body burden was lost per day in both *Hydropsyche* and *Arctopsyche*. The rate constants of Cu loss were not significantly different (k_e_ = 0.17 ± 0.01 d^-1^ and 0.18 d^-1^ ± 0.04 d^-1^, respectively; ANCOVA, *P* = 0.17, Fig 2). Likewise, the rate constants of Cd loss were not significantly different (*Arctopsyche* k_e_ = 0.14 ± 0.06 d^-1^, *Hydropsyche* k_e_ = 0.24 ± 0.01 d^-1^; ANCOVA, *P* = 0.93, Fig 2).

### 3.3. Determination of the relative importance of aqueous and dietary exposure pathways

The experimentally derived conditional rate constants in Table 2 were used in Eq 3 to compare predicted vs observed Cd and Cu caddisfly concentrations at sites in the Clark Fork River (CFR), a large metal-impacted Superfund site in western Montana (Table 3). Long-term monitoring studies in this river have used caddisflies as biomonitors of environmental condition, linking changes in metal concentrations to remediation efforts [6, 31, 49–53]. Site-specific dissolved metal concentrations, ([M]_w_) were adjusted to 10% of total dissolved Cu concentrations and 80% total dissolved Cd concentrations to account for complexation by inorganic ligands [43]. Dietary metal concentrations, [M]_food_ was determined from material in caddisfly foreguts (a compartment that likely represents recently ingested material) collected at the co-occurring sites (Table 3). Tissue concentration predictions were made for aqueous exposure alone (thus ignoring diet) and for aqueous plus dietary exposure.

**Table 3:** Biogeochemical data from the Clark Fork River, Montana, provide the basis for site-specific predictions of Cu and Cd concentrations in two species of caddisflies (Fig 3). Dissolved concentrations, [M]w, are based on seasonal (May-August) averages and coincide with the life-history of the caddisfly larvae; dietary exposure, [M]f, was determined by analyzing metal concentrations in caddisfly foreguts, a proxy for dietary exposure (see methods for more detail). Species do not always co-occur at each site (--).

USGS Site Number	Citation (tissue and dissolved data)	*[Cu]w μg/L	*[Cd]w μg/L	[Cu]f μg/g (*Hydropsyche)*	[Cu]f μg/g (*Arctopsyche)*	[Cd]f μg/g (*Hydropsyche)*	[Cd]f μg/g (*Arctopsyche)*
12334550	46	0.48	0.02	212 ± 9	130 ± 42	1.3 ± 0.1	2.2 ± 0.9
12323800	47	0.88	0.05	580 ± 256	--	4.4 ± 2.2	--
12324680	47	1.2	0.06	716 ± 119	--	2.7 ± 0.7	--
12323800	48	0.4	0.02	825 ± 342	--	4.7 ± 2.8	--
12334550	48	0.32	0.02	153 ± 64	--	1.8 ± 0.5	--
12340500	48	0.18	0.01	123 ± 57	--	2.6 ± 1.1	--
12323800	49	0.32	0.02	541 ± 233	--	4.3 ± 1.8	--
12324680	49	0.4	0.02	--	139 ± 47	--	6 ± 2.9
12334550	49	0.22	0.02	121 ± 52	125 ± 102	1.2 ± 0.4	1.3 ± 0.4
12340500	49	0.14	0.01	109 ± 28	88 ± 33	2.2 ± 1.2	2.1 ± 1.1
12323800	50	0.43	0.04	369 ± 116	--	3.6 ± 0.2	--
12324200	50	0.87	0.05	319 ± 153	--	2.8 ± 1.7	--
12324400	50	0.91	0.06	355 ± 125	247 ± 62	2.6 ± 0.4	5.3 ± 0.4
12324680	50	0.65	0.03	194 ± 27	126 ± 8	3.7 ± 0.8	5.5 ± 1.6
12331800	50	0.52	0.03	175 ± 42	130 ± 35	1.7 ± 0.1	3.3 ± 1.3
12334550	50	0.28	0.02	95 ± 16	109 ± 2	1.2 ± 0.1	1.8 ± 0.4
12340500	50	0.18	0.02	123 ± 54	100 ± 16	1.4 ± 0.5	2.5 ± 0.7

*Dissolved concentrations are 10% of total dissolved Cu and 80% of total dissolved Cd to account for complexation by inorganic ligands [43].

**Fig 3:**
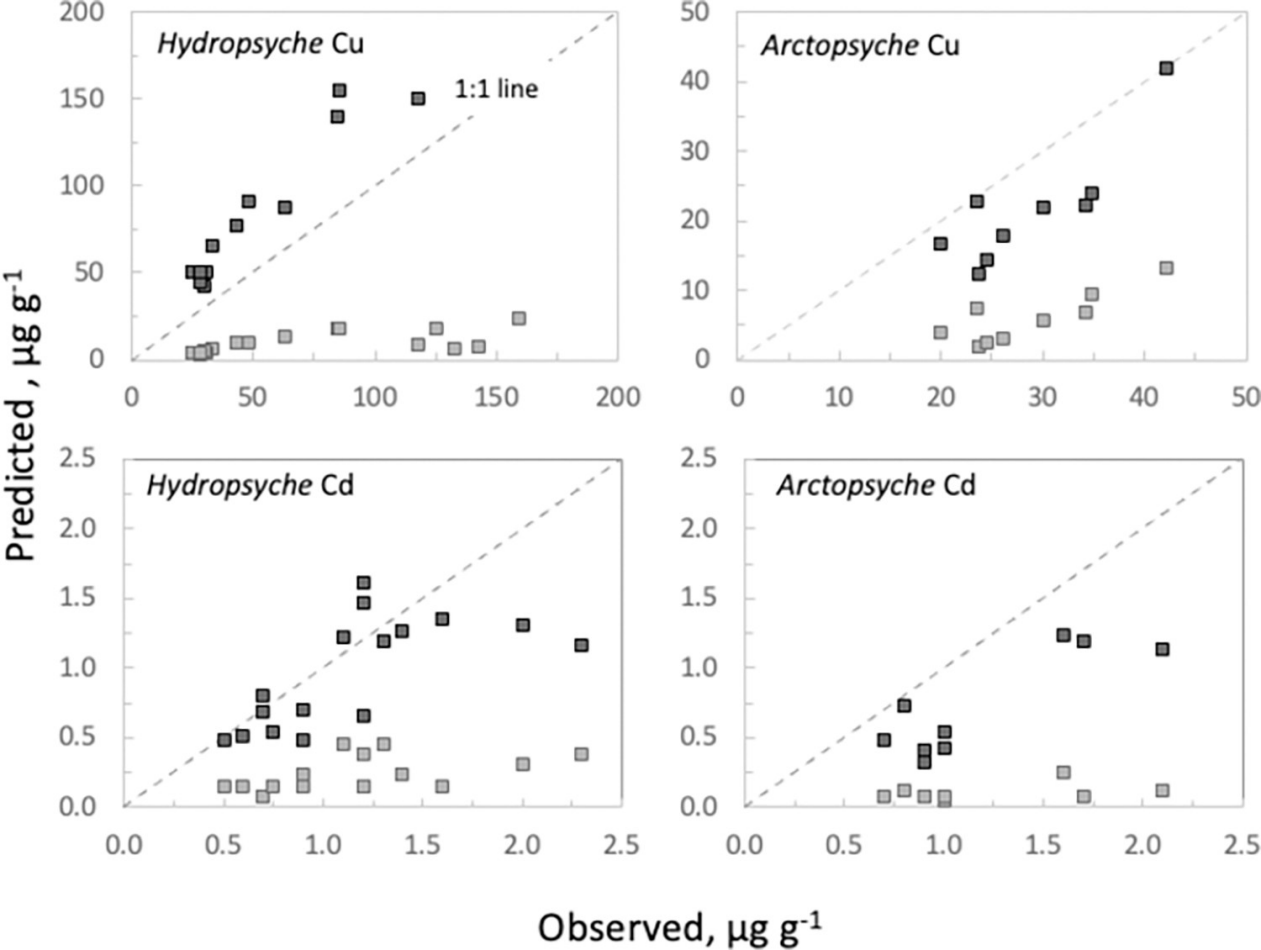
Biodynamic model predictions are plotted against observed Cu and Cd concentrations for *Hydropsyche* and *Arctopsyche*. Predictions based on the laboratory derived parameters (Table 2) and estimates of site-specific dissolved and dietary exposure (Table 3). Observed values are plotted against “dissolved only exposure predictions” (light squares) and “dissolved + dietary exposure predictions” (dark squares, Eq 3). Dashed line represents the 1:1 relationship.

Based on “dissolved only” exposure conditions, predicted Cu concentrations in both species of caddisflies accounted for only 15–20% of observed concentrations (Fig 3) and thus largely underestimate what is observed in nature. Keeping the rate constants the same, but including dietary exposure increases the predicted tissue Cu concentrations to 70–175% of observed concentrations (*Hydropsyche* exceeding observed by 75%) (Fig 3). Variability in food type, food quality, AE and IR may account for the over (or under) estimated values.

Predictions for Cd in caddisfly tissue are similar to those observed for Cu: dissolved only exposures contribute between 10–35% but with dietary exposure increase to 60–90% of observed tissue concentrations. Unlike Cu, predicted *Hydropsyche* Cd does not systematically exceed the observed values, but variability associated with predicted values is likely driven by the same factors mentioned above. Field-collected data are inherently variable, given the complex environmental conditions that occur in nature. However, this exercise demonstrates the utility of approximating bioavailable Cu and Cd when exposure parameters are known.

## 4. Discussion

Bioassessment studies use the occurrence and distribution of aquatic insects to provide evidence of impairment or recovery in contaminated waterways. In this approach, causal linkages to contaminants such as metals are inferred. However, biomonitoring can be strengthened by understanding the mechanisms of metal tolerance and species sensitivity. The caddisfly biodynamic model presented here integrates site-specific environmental factors with species-specific biological controls on metal bioaccumulation and provides insight into how two common biomonitors respond to, and integrate, environmental metals exposure.

### 4.1 Species-specific influx and efflux

The biokinetic parameters described here fall within range of other aquatic insects, including mayflies [22] and stoneflies [54]. If species are phylogenetically related, they can express comparable traits (e.g., biokinetic parameters), perhaps as an evolutionary strategy in response to detoxifying metals [26]. *Arctopsyche* and *Hydropsyche* here are phylogenetically similar (both are filter-feeding caddisflies and belong to the family Hydropsychidae) and co-occur in metal-impacted rivers. But where they co-occur, Cu and Cd tissue concentrations can vary [5], with *Hydropsyche* having up to 50% higher Cu concentrations, and *Arctopsyche* having up to 2-fold higher Cd concentrations.

In this study, rate constants for the dissolved Cu influx and efflux were not significantly different between the two species and does not appear to be a factor contributing to bioaccumulation differences observed in nature (Table 2). And while dissolved Cd influx is 3-fold faster in *Hydropsyche*, the biodynamic model predictions indicate that dissolved Cd accounts for <20% of the total tissue body burden in both species (Fig 3). Similar biokinetic studies for *Arctopsyche* could not be found for comparison, but the findings presented here for *Hydropsyche* are consistent with other investigators. Studies using *Hydropsyche oslari* and *Hydropsyche californica* reported Cd influx and efflux rates similar to this study [32, 55]. Copper biokinetic results in Evans et al., [33] reported a Cu uptake rate constant for *Hydropsyche betteni* at 0.62 L g^-1^ d^-1^ ± 0.15, a rate ~5-fold lower than findings in this study and Cu efflux of 0.08 ± 0.01 d^-1^, about half of what is reported here (methodological differences such as a longer exposure/sampling interval and water hardness may contribute to this difference).

### 4.2 Physiological differences and the importance of diet

In this study, predicted tissue concentrations based solely on dissolved exposures accounted for ~10–20% of the Cu and Cd body burden of field collected organisms (Fig 3). This explains, in part, the disconnect between environmental dissolved concentrations that are below the chronic exposure criteria for aquatic life and high tissue body burdens measured in caddisflies. For example, average dissolved concentrations in the Clark Fork River (CFR), a river impacted by over a century of copper mining, range from 4–10 μg L^-1^ Cu and 0.03 to 0.08 μg L^-1^ Cd [53]. These dissolved concentrations are lower than the chronic exposure criteria for aquatic life for the CFR (13 μg L^-1^ Cu and 0.36 μg L^-1^ Cd, [56]) and bioavailability is likely further reduced by metal complexation with organic and inorganic ligands [57]. Yet under these conditions, tissue concentrations in *Hydropsyche* and *Arctopsyche* in the CFR are up to 5-fold higher than organisms collected from uncontaminated CFR tributaries [53], supporting the finding that metal uptake is occurring primarily through the diet.

When diet was included in the caddisfly biodynamic model, predicted Cu and Cd tissue concentrations in both species increased to 60–88% of observed values, indicating that diet is the predominant route of Cu and Cd exposure. Dietary exposure is influenced by food type and associated metal concentration ([M_f_]), AE, and IR. These factors can be highly variable, especially in nature, so to understand the applicability (and limitations) of the model, each parameter is discussed in detail below.

### Food type

Caddisflies are omnivorous filter-feeders with a diet consisting of suspended particulate organic material (POM) that generally consists of diatoms, detritus, smaller insects [58]. The diet will vary with instar, season, and resource availability. In this study, dietary exposure ([M_f_]) was characterized using foregut metal concentrations as a surrogate for dietary metals exposure. At sites where both species co-occur (Table 3), average Cu dietary concentrations in *Hydropsyche* were generally higher than *Arctopsyche* (216 ± 181 μg g^-1^,132 ± 46 μg g^-1^, respectively). At the same sites, average Cd concentrations in the diet of *Arctopsyche* were higher than *Hydropsyche* (3.3 ± 1.8 μg g^-1^ vs 2.0 ± 0.8 μg g^-1^, respectively). Field data are inherently variable, but this finding suggests that [M_f_] parameter may explain, at least in part, the tissue body burdens observed in nature.

### Food quality

Metal bioavailability is affected by the presence of organic material [18, 59, 60] but inorganic material will also contribute to total dietary metal concentrations used in the model. Because filter-feeding caddisflies likely capture and ingest inorganic material as part of their feeding behavior, consideration of the inorganic component is important. Cain et al. [61] reported high Cu AE (>70%) by benthic grazers that ingested periphyton enriched with synthetic colloidal hydrous ferric oxide, demonstrating that inorganic material can influence Cu bioavailability. The caddisflies in this study were fed laboratory cultures of snail tissue (*Arctopsyche* experiment) and worms (*Hydropsyche* experiment), resulting in AEs between 70–96%. The Aes in these controlled experiments are likely higher than what occurs in nature where organisms are exposed to a more heterogenous diet and were not meant to simulate natural conditions in the field.

### Physiological factors (IR, growth)

In this study, Irs for both species were ranged from 0.03–0.07 g g^-1^ d^1^, but in nature, late-instar caddisflies can ingest up to 10–15% of their body weight per day [62]. McCullough et al. [63] reported a mean ingestion rate for *Hydropsyche occidentalis* at 0.18 g g^-1^ d^1^, more than two-fold higher than Irs reported here. Lower estimates of IR will under-predict tissue body burdens, and may explain, in part, the lower predicted values in this model (Fig 3). The Irs from this study are consistent with rates reported for aquatic Insects in other studies [e.g., 22, 54], but iRs determined in laboratory studies may underestimate iRs in nature. Net-spinning caddisflies are dependent on unidirectional currents for food capture and though the experimental set-up for this study included directional currents, the iRs for this model represent a conservative estimate of what occurs in nature.

The influence of growth and metal dilution are additional factors that can influence the model outcome. Because the body weight of these organisms remained consistent during these short-term experiments [38], the effect of growth (k_g_) on metal tissue concentrations can be ignored for the purposes presented here. And while caddisfly growth rates can be highest during the warmer months (one instar change in 30 days, [64]), any dilution due to increasing growth may be offset by a corresponding increase in IR [62].

### 4.3 Caddisfly model applications

Caddisflies have been successfully used to identify spatiotemporal trends in metal contaminated rivers [3, 5, 6, 65, 66], but interpretation regarding exposure history is limited without an understanding of how biokinetics control tissue residence time. That is, how best to interpret metal concentrations in the context of exposure once the organism is collected. The parameters and biodynamic model presented here provide the basis for assessing tissue residence time and exposure history of bioaccumulated Cu and Cd in *Hydropsyche* and *Arctopsyche*.

**Fig 4:**
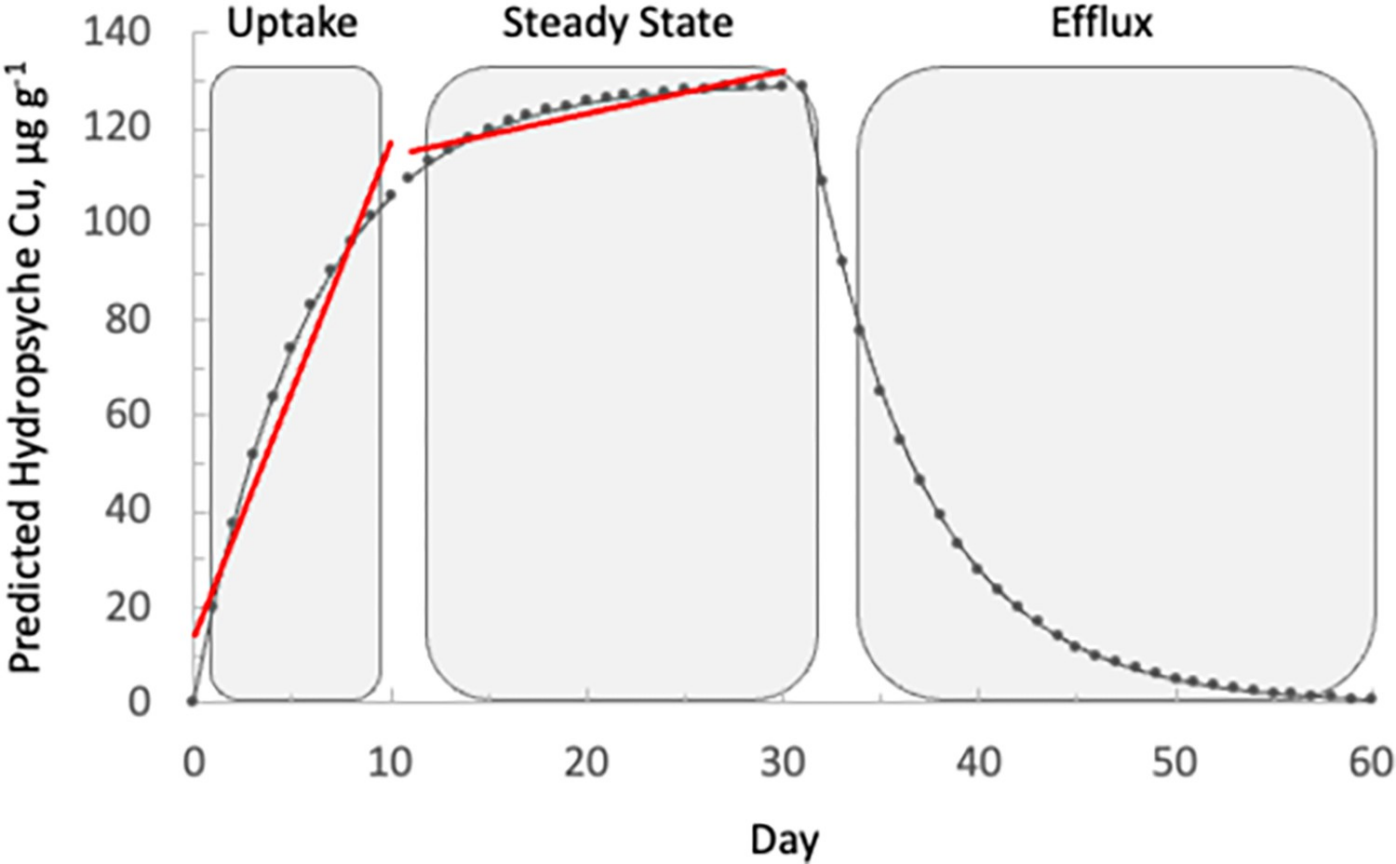
Predicted Cu concentration in *Hydropsyche* over time. Shaded areas represent the period of uptake (Eq 2) and efflux (Eq 8). Piecewise regression (red solid lines) was used to identify the period of uptake and near steady state.

A hypothetical Cu exposure history curve was developed using experimentally derived parameters discussed here (Eq 2, Fig 4) and environmental concentrations (dissolved and diet) from field samples (Table 3). Although this example is not intended to duplicate natural conditions, it provides a foundation for better understanding the relationship between metal bioaccumulation and exposure history. Assuming physiological rate constants of Cu uptake and loss remain stable over time and dissolved, and dietary Cu exposure also remain constant (using average dissolved and dietary Cu concentrations for *Hydropsyche* presented in Table 3), the model indicates that *Hydropsyche* can accumulate ~90% of its body burden in 10 days and reach steady state by day 30 (piecewise regression, *P* = 0.03) (Fig 4). When the Cu exposure terms in Eq 2 are removed, (a scenario unlikely to occur in nature but useful for the purpose of evaluating efflux over time), Cu concentrations in *Hydropsyche* decline to <10% of the steady state concentrations in ~14 days (Eq 8, Fig 4). While this part of the curve is driven solely by the efflux rate, k_e_ (17% loss of Cu per day), it demonstrates the importance of considering biokinetics when interpreting metal biomonitoring data. In nature, rapid efflux may be offset by chronic exposure to elevated metal concentrations, so sampling during base-flow conditions, when variability in flows (and associated resource subsidies) is minimal, may improve the accuracy of interpreting tissue body burdens in metal contaminated rivers.

### 4.4. Model parameter uncertainties

The dissolved uptake rates (k_u_) reported here contribute only a small proportion towards total tissue body burden, a finding similar to rates reported in other studies [22, 61]; thus, variability associated with this rate constant is likely negligible. Copper and Cd efflux rates (k_e_) in these caddisflies are also similar to other metal tolerant aquatic insects that are known to be relatively metal tolerant [22] indicating that experimental loss rates may reasonably estimate what occurs in nature. While growth rates, which were not part of this study, could influence tissue body burden, the presence of continued dietary metal exposure reduces the likelihood that growth would have a dominant affect. The largest uncertainty in the model is in characterizing natural dietary exposure ([M_f_]), and physiological rate constants of AE and IR. Dietary exposure will integrate species traits that vary according to life-history patterns and resource availability [64, 67]. Food type and quality will influence AE and likely vary over the lifespan of the organism. The ingestion rates reported here are probably lower than rates under natural conditions. Also, the rates may vary with changes in dietary preference for late instar caddisflies. While the model can be refined to include more complex environmental and physiological behavior (which falls outside the scope of this study), it does provide evidence that dissolved Cu and Cd exposure only minimally influences caddisfly tissue body burdens and establishes a baseline for characterizing exposure history and tissue residue time.

### 4.5 Implication of biokinetics to biomonitoring programs

The results presented here build upon a very limited data set for other freshwater bioindicators [17, 22, 32–33, 54–55], and help elucidate the processes that contribute to the effective application of aquatic invertebrates as bioindicators.

Dissolved Cu and Cd uptake in these caddisflies are similar to rates reported for some heptageniid mayflies, but about 4-fold lower than some ephemerellids [22]. Differences may be due to variation in the surface area to volume ratio between organisms, which enhance the diffusion and transport of metals or species-specific osmoregulatory function [55]. But despite the relative differences in metal uptake rates, dissolved exposure alone greatly underestimates tissue body burdens in both *Hydropsyche* and *Arctopsyche* (Fig 3). These findings are supported by other studies, which demonstrate the importance of dietary exposure and metal bioaccumulation [22]. And while water quality criteria are a common measure of stream health, dissolved Cu and Cd concentrations may under predict caddisfly metal bioaccumulation and underestimate ecological risk.

These caddisflies have a relatively rapid Cu and Cd efflux rate (~20% per day) which falls within the range measured for mayflies [22] but an order of magnitude faster than Cd efflux in stoneflies [54]. A rapid loss rate indicates that a species can respond rapidly to fluctuations in environmental metal exposures. Thus, tissue concentrations in these caddisflies likely represent a 2–3-week exposure period at the time of collection. If biomonitoring programs establish goals that are consistent with the biological mechanisms controlling physiological uptake and loss of these organisms, bioaccumulation data will have temporal context. For example, because discharge is the primary mechanism of metals transport in a lotic system, flow conditions can be a proxy for metals exposure [6]. Sampling during base flow conditions will likely coincide with steady state for these biomonitors given that site-specific conditions have likely remained constant for a sustained period. Alternatively, the rapid efflux of these caddisflies suggests that they can also be used to identify episodic events because they can respond relatively quickly to changes in environmental metals exposure (Fig 4). The species-specific physiological rate constants described here will help strengthen bioaccumulation field studies by providing a foundation for interpretating biological response to changing environmental conditions.

## 5. Conclusions

Long-term field studies demonstrate the utility in using both *Hydropsyche* and *Arctopsyche* as indicators of bioavailable metal exposure in mine-impacted streams. But interpretations related to exposure history are limited without an understanding of the biokinetics that control metal uptake and loss. This study identified the biokinetic parameters necessary to link bioaccumulation data from field studies to environmental exposure conditions. While both species are metal tolerant and can withstand relatively high dissolved concentrations of Cu and Cd in short-term laboratory experiments, dissolved metal uptake represents <20% of their Cu and Cd body burden and indicates that the primary mode of metal accumulation is through dietary uptake. There were no species-specific differences in dissolved uptake or efflux, but *Hydropsyche* had a higher AE and faster IR than *Arctopsyche*. While these differences may be due to how each species tolerates laboratory conditions, these physiological parameters fall within range of values determined for other aquatic taxa. The relatively fast efflux rates (k_e_) for both caddisflies are traits that constrains metal accumulation and enhances their metal tolerance. Application of the biodynamic model using the parameters derived in this study suggests that these species can reach steady state within <30 days and can lose 90% of their body burden in ~2 weeks if exposure is removed. And while these parameters may vary slightly due to changes in life-history traits (e.g., growth, food selectivity), this study presents some boundaries for each parameter and identifies dietary exposure as the primary source of metal to these species. Understanding the physiological factors affecting metal uptake and loss increases the utility of these biomonitors, as measures for both short-term (weeks) and long-term (decadal) biomonitoring programs and furthers the understanding related to biological uncertainties associated with bioaccumulation studies.

## Supporting information

S1 Fig Experimental depuration chamber for dietary exposure experiment. Fecal material from each individual caddisfly was collected in a container attached behind each retreat.
(TIF)
