# Supplementary figures and images for: A biodynamic model predicting copper and cadmium bioaccumulation in caddisflies: Linkages between field studies and laboratory exposures
Source: PLoS One. 2024 Feb 22;19(2):e0297801. doi: 10.1371/journal.pone.0297801 (PMC10883540; doi:10.1371/journal.pone.0297801)

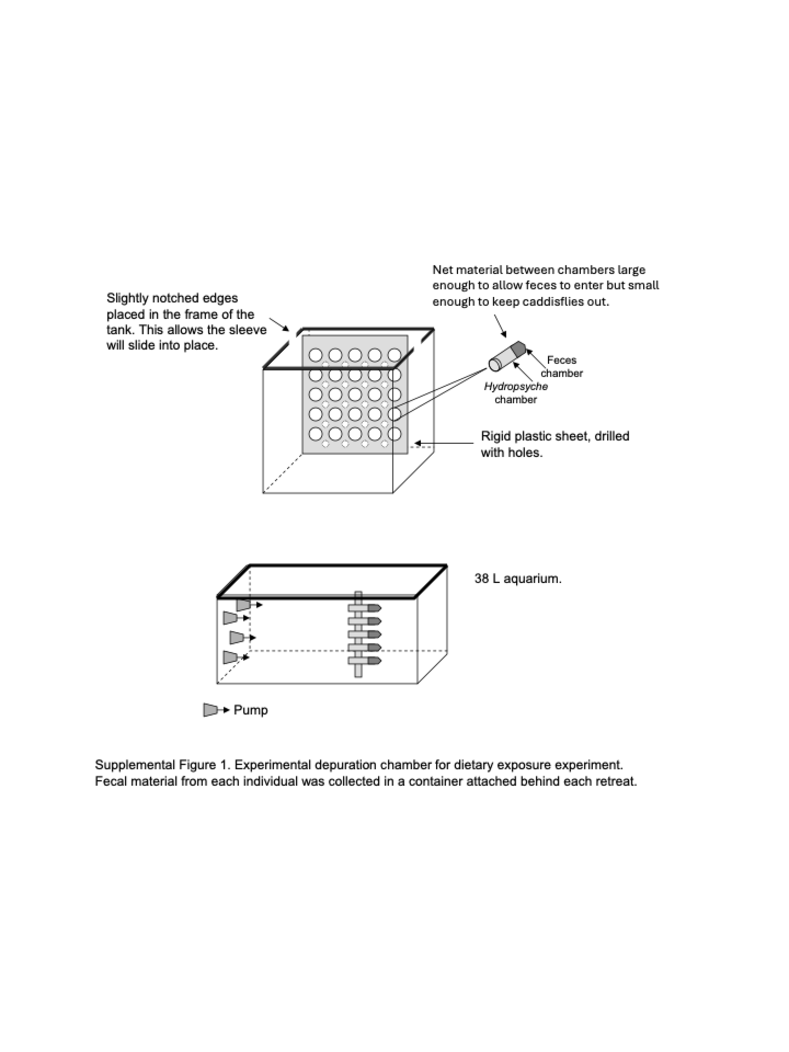

Supplement: S1 Fig — Fecal material from each individual caddisfly was collected in a container attached behind each retreat. (TIF) [file pone.0297801.s001.tif]
